# Case report: Clinically mild encephalitis/encephalopathy with a reversible splenial lesion: an autopsy case

**DOI:** 10.3389/fneur.2023.1322302

**Published:** 2024-01-04

**Authors:** Maho Hayashi, Midori Ueda, Koji Hayashi, Ei Kawahara, Shin-ichiro Azuma, Asuka Suzuki, Yuka Nakaya, Rei Asano, Mamiko Sato, Toyoaki Miura, Hiromi Hayashi, Kouji Hayashi, Yasutaka Kobayashi

**Affiliations:** ^1^Department of Diabetes and Endocrinology, Fukui General Hospital, Egami-cho, Fukui, Japan; ^2^Department of Rehabilitation Medicine, Fukui General Hospital, Egami-cho, Fukui, Japan; ^3^Department of Pathology, Fukui General Hospital, Egami-cho, Fukui, Japan; ^4^Department of Rehabilitation, Faculty of Health Science, Fukui Health Science University, Egami-cho, Fukui, Japan

**Keywords:** clinically mild encephalitis/encephalopathy with reversible splenial lesions, corpus callosum splenium, autopsy: MRI, hypoglycemia, cytotoxic edema

## Abstract

Clinically mild encephalitis/encephalopathy with a reversible splenial lesion is a clinicoradiological syndrome characterized by transient neuropsychiatric symptoms and hyperintensity of the splenium of the corpus callosum on diffusion-weighted MRI. Although intramyelinic edema and inflammatory cell infiltration can be predicted by MRI, the pathology of the splenium of the corpus callosum remains unknown. We encountered a case of clinically mild encephalitis/encephalopathy with a reversible splenial lesion and hypoglycemia in a patient who died of sepsis, and an autopsy was performed. The postmortem pathological findings included intramyelinic edema, myelin pallor, loss of fibrous astrocytes, microglial reactions, and minimal lymphocytic infiltration in the parenchyma. Based on these findings, transient demyelination following cytotoxic edema in the splenium of corpus callosum was strongly considered a pathogenesis of “clinically mild encephalitis/encephalopathy with a reversible splenial lesion” associated with hypoglycemia, and it could be generalized for the disease associated with the other causes. As cytotoxic edema could be the central pathology of the disease, the recently proposed term cytotoxic lesions of the corpus callosum may be applicable to this syndrome.

## Introduction

Clinically mild encephalitis/encephalopathy with a reversible splenial lesion (MERS) is a clinicoradiological syndrome that can be characterized by the onset of reversible neuropsychiatric symptoms followed by the MRI hyperintensity in the splenium of corpus callosum (SCC) frequently spreading to the adjacent deep white matter (1–3), which is caused by a variety of infectious and non-infectious conditions, including antiepileptic drug toxicity, hypoglycemia, or hypernatremia (4, 5). The syndrome is also called reversible splenial lesions (6), reversible splenial lesion syndrome (RESLES) (7, 8), or cytotoxic lesions of the corpus callosum (CLOCCs) (9). Although the pathogenesis of the syndrome, in any case, is still unknown, what occurs in the splenium of the corpus callosum is predicted to be intramyelinic edema due to the separation of myelin layers and inflammatory cell infiltration (2, 10, 11) based on MRI. However, there are no previous reports on the histopathological findings of MERS. In this study, we describe the postmortem histopathological findings of a case of MERS associated with hypoglycemia and discuss the pathogenesis with clinicoradiological assessments.

## Case presentation

### Clinical course

**Figure 1:**
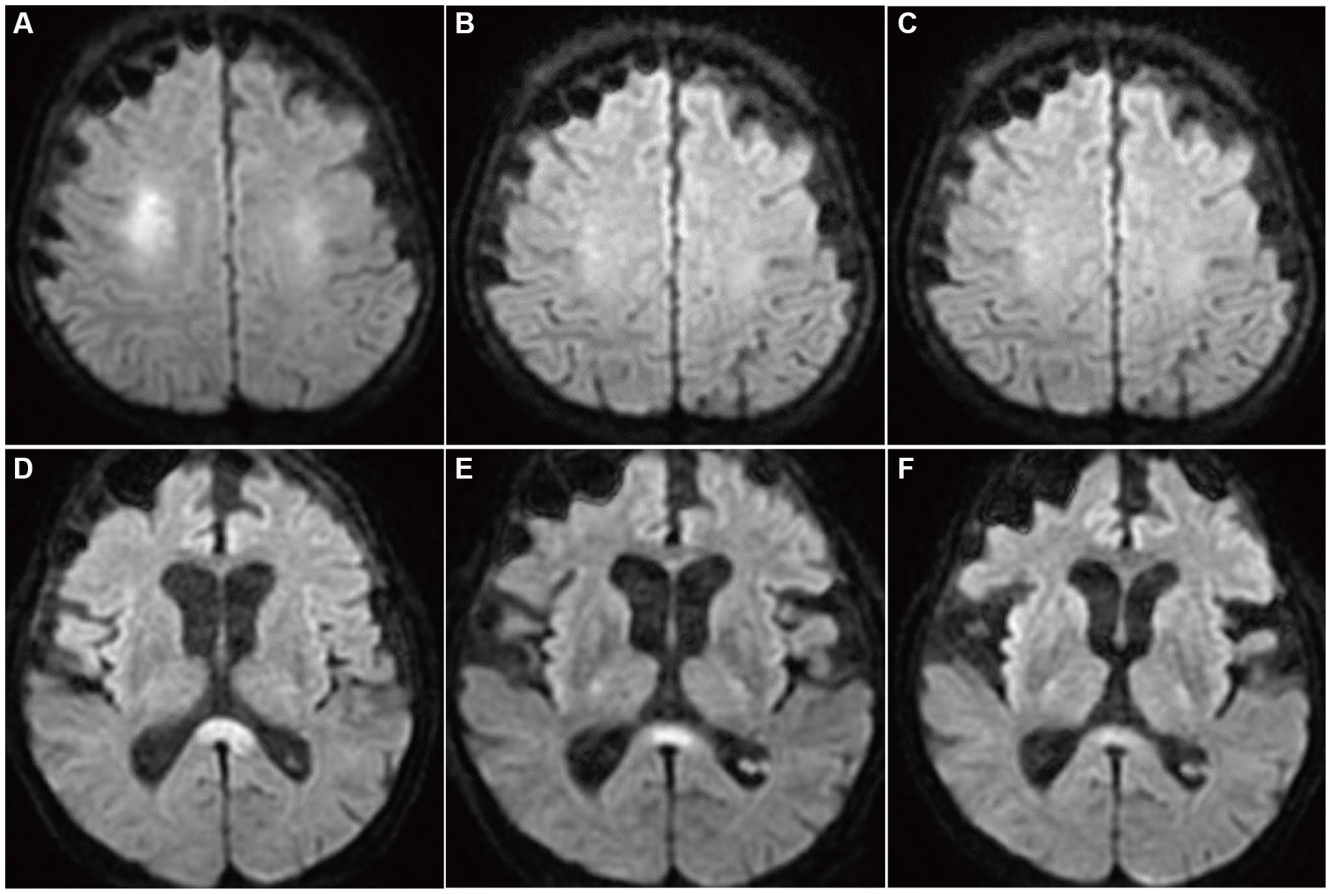
Diffusion-weighted brain MRIs (DWI). On day 2 of admission, hyperintensity was noted in the bilateral deep white matter and the corpus callosum splenium **(A,D)**. On days 3 **(B,E)** and 6 **(C,F)**, the hyperintensities were attenuated gradually.

**Figure 2:**
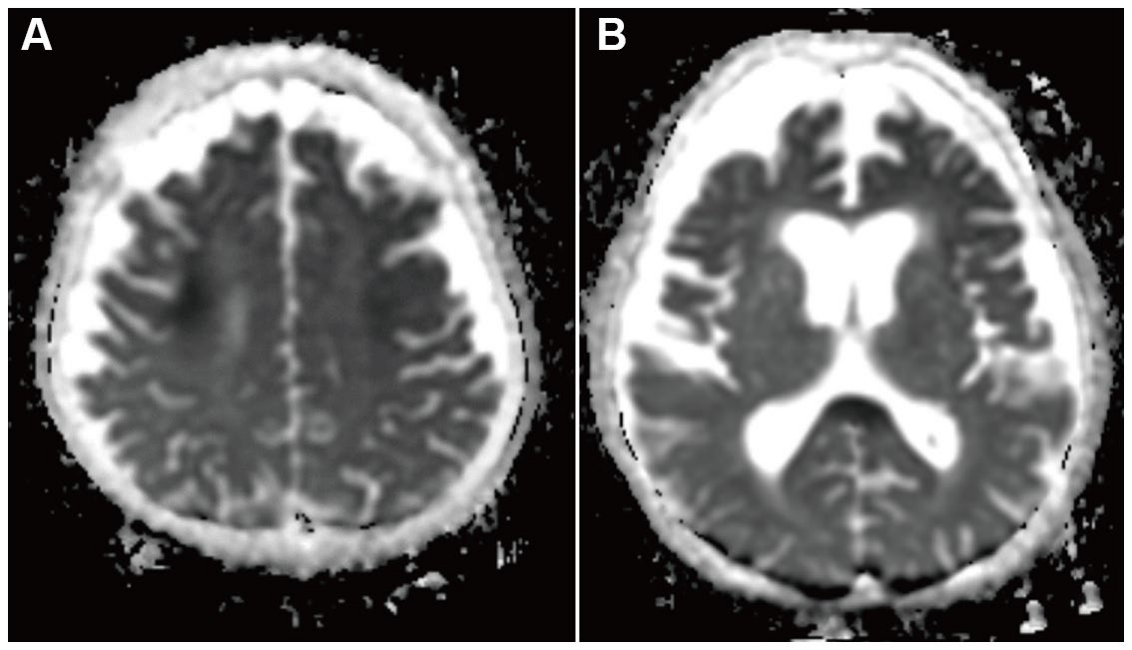
Apparent diffusion coefficient maps brain MRI on day 2 of admission. Hypointensity was in the bilateral deep white matter **(A)** and the corpus callosum **(B)**. This hypointensity was consistent with DWI hypointensity areas.

A 68-year-old man who had been treated with olanzapine for schizophrenia and obsessive-compulsive disorder presented with disturbance of consciousness (DOC). He had a history of hypothyroidism, dementia, and sigmoid colon volvulus. He had been placed in the psychiatry department for a long time and had severe constipation and ileus. Blood tests have shown mild hyponatremia (120–130 mmol/L) for several years. Fasting blood sugar levels (64–87 mg/dL) were in the reference range (60–109 mg/dL). His medications had not changed through the years. Four days before he was transferred to our hospital, he had been unable to eat, and the DOC appeared. As the DOC persisted without improvement, the patient was transferred to our hospital by ambulance. At admission, his body height was 168 cm, body weight was 45.8 kg, body temperature was 36.4°C, blood pressure was 115/90 mmHg, pulse rate was 74/min, and SpO_2_ was 98% (O_2_ 2 L, nasal). We noticed normal heart and lung sounds and hypoactive bowel and tympanitic sounds in the abdomen. Neurological findings showed that the Glasgow coma scale (GCS) score was E1V1M1, pupil diameter was 2.0/2.0, light reflex was prompt bilaterally, oculocephalic reflex was normal, involuntary and autokinetic movements were absent, deep tendon reflex was normal in the upper limbs but decreased in the lower limbs, and the pathologic reflex was absent. Blood examinations revealed mild anemia, severe hypoglycemia (13 mg/dL), mild hepatic function disorder, mild hyponatremia (125 mmol/L), and mildly elevated C-reactive protein levels. Endocrine tests showed no abnormalities except for the already-known hypothyroidism. He underwent glucose and natrium replacement therapy, and blood sugar levels (80–142 mg/dL on day 2 to day 6) and serum natrium levels (129–145 mmol/L on day 2 to day 6) were improved. However, the DOC did not improve. Diffusion-weighted brain magnetic resonance imaging (MRI) on day 2 of hospitalization showed hyperintensity in the symmetrical cerebral white matter and corpus callosum (Figures 1A,D). Brain MRI apparent diffusion coefficient (ADC) images showed hypointensity in the areas consistent with hyperintensity by diffusion-weighed images (Figures 2A,B). Brain MRI was performed on days 3 and 6, which revealed gradual attenuation of hyperintensity in these areas (Figures 1B,C,E,F). Abnormal blood sugar levels and MRI hyperintensity improved, but the DOC remained unchanged for several days. On day 17, the GCS score improved to E1V2M5. On day 27, he complained of abdominal bloating, and the food supply was stopped. Owing to malnutrition and poor general condition, anemia progressed, hypothermia and hypotension began on day 44, and the patient died on day 48. Consent for postmortem autopsy, including that for craniotomy, was obtained.

### Postmortem pathology

At autopsy, the body was emaciated, and histological examination showed that the skeletal muscle cells and adipocytes were remarkably atrophic throughout the body. The heart (160 g) and liver (590 g) exhibited brown atrophy. The thyroid gland was markedly atrophic (4 g) with multiple fibrotic foci. The anasarca was marked (ascites, 2,200 mL; pleural effusion, 1,950 mL; and 1,950 mL). Malnutrition was considered marasmic kwashiorkor because of repeated ileus and severe diarrhea. Acute pyelonephritis, bronchopneumonia, and acute splenitis were further identified, and sepsis was suggested as the final cause of death.

**Figure 3:**
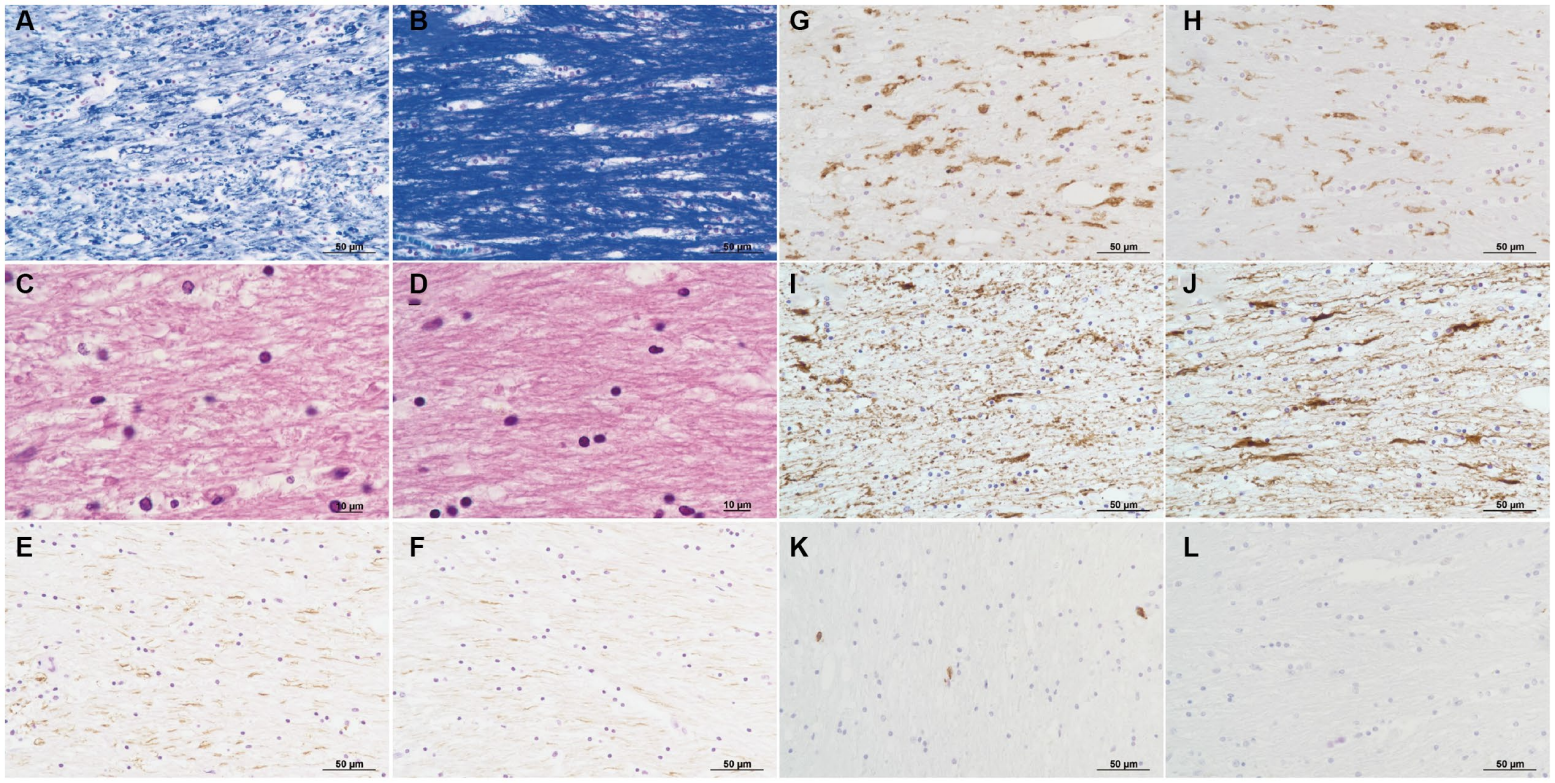
Pathology of the corpus callosum splenium. Pathological changes in the area of myelin pallor **(A,C,E,G,I,K)** are compared to those in the unaffected area of myelin unpallor **(B,D,F,H,J,L)** in corpus callosum splenium. Klüver-Barrera stain **(A,B)**. Myelin is much paler **(A)** than that in the unaffected area **(B)**. H&E stain **(C,D)**. Intramyelinic edema is noticed **(C)**. Immunoperoxidase for neurofilaments **(E,F)**. Axons with phosphorylation are clearly visible **(E,F)**. Immunoperoxidase for HLA-DR **(G,H)**. Microglia increase in number and size **(G)**. Immunoperoxidase for glial fibrillary acidic protein (GFAP) **(I,J)**. Fibrous astrocytes decrease in number **(I)**. Immunoperoxidase for CD8 **(K,L)**. CD8-positive lymphocytes are infiltrated **(K)**.

**Figure 4:**
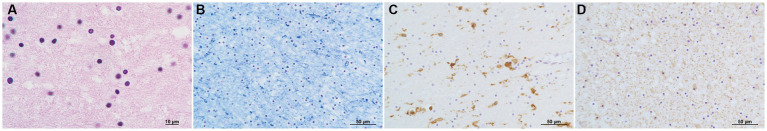
Pathology of the deep white matter. H & E stain **(A)**. Vacuolization is marked. Klüver-Barrera stain **(B)**. Myelin is pale. Immunoperoxidase for HLA-DR **(C)**. Microglia increase in number and size. Immunoperoxidase for GFAP **(D)**. Astrocytes decrease in number.

No remarkable macroscopic changes were observed in the brain. Microscopic examination revealed several pathological changes in the SCC. Klüver-Barrera staining showed uneven myelin pallor in the SCC (Figure 3A) and deep white matter. All findings around the myelin pallor (Figures 3A C E G I K) were compared with those in the areas without myelin pallor (Figures 3B D F H J L) in the SCC. In the area of the myelin pallor, eosin staining for myelin showed pallor as well as vacuolization in myelin sheaths (Figure 3C). Pale myelin and vacuoles were negative for periodic acid-Schiff (PAS) and Alcian blue staining, indicating simple intramyelinic edema. Immunohistochemical analysis showed that neurofilaments were preserved in the myelin sheaths and were more clearly positive (Figure 3E) than in the regions without pallor (Figure 3F), possibly due to myelin loss. Microglia evaluated by the anti-HLA-DRA antibody was in the activated form with swollen cytoplasm and increased in number (Figure 3G). In contrast, some astrocytes evaluated by the anti-glial fibrillary acidic protein (GFAP) antibody were lost (Figure 3I) in the demyelinated area. CD8-positive lymphocytes were scattered throughout the parenchyma (Figure 3K). In the white matter of the cerebrum, Klüver-Barrera staining also showed myelin pallor (Figure 4A). Vacuolization was marked (Figure 4B), and immunohistochemical studies also showed preserved myelin sheaths, active microglia (Figure 4C), and a reduction of astrocytes (Figure 4D) in the white matter. No other abnormalities were observed in the brain.

## Discussion

To the best of our knowledge, this is the first report to describe the pathological findings of MERS. Histopathological findings revealed intramyelinic edema, myelin pallor, microglial reactions, loss of fibrous astrocytes, and minimal infiltration of lymphocytes into the SCC. As axons are well preserved in the area of myelin pallor, we may use the term demyelination, even if it is transient. Demyelination is transient when the causal stimulation is removed (12). Notably, in our case, the evidence of demyelination persisted in the postmortem brain 6 weeks later, when the abnormal MRI signal decreased.

It was originally considered that MERS occurs subsequent to infectious disease, especially in children, but previous reports mention various diseases related to MERS; hypoglycemia was listed as the cause of MERS (5). Furthermore, several cases were not recognized as MERS, whereas they were recognized as a specific condition (13–15), as exemplified by the case Maruya et al. (16) reported. The report showed that brain MRI with hypoglycemia showed hyperintensity in the splenium of the corpus callosum and bilateral posterior limbs of the internal capsules. The case was not documented as MERS but met the diagnostic criteria for MERS based on a nationwide survey on the epidemiology of acute encephalopathy in Japan (1). Kang et al. (17) reported 11 cases of diffusion-weighted MRI findings of hypoglycemic encephalopathy, and many cases presented with hyperintensity in the corona radiata; however, there was no case of abnormal signals in the corpus callosum (17). These hyperintensities on diffusion-weighted MRI in the brain are cytotoxic edema, and it is estimated that the mechanism of diffusion restriction in the brain by hypoglycemic encephalopathy is caused by not only acute ischemia but also hypoglycemia itself. *In vivo* animal model studies have demonstrated diffusion alterations due to hypoglycemia (18, 19). In addition, hyponatremia can be related to MERS. Takanashi et al. (4) reported that blood natrium in patients with MERS was significantly decreased compared to that in controls. Our patient was diagnosed with hypoglycemia and hyponatremia on admission, both of which we believe were associated with MERS. However, it is likely that the main cause of MERS in the present case is not hyponatremia but hypoglycemia, because the levels of mild hyponatremia have remained unchanged through the years, including the duration of the event of MERS. Moreover, Nelles et al. (20) reported a schizophrenic case with a transient splenium lesion treated with olanzapine and citalopram. They presumed that olanzapine interferes with sodium metabolism, leading to severe toxicity states in the SCC (20). Like the previous case, ours might have been toxic states in the SCC from the long-term administration of olanzapine.

The pathogenesis of MERS was unknown, but it was widely hypothesized that an inflammatory process involving cytokines triggers glutamate accumulation in the extracellular space, resulting in cytotoxic edema, particularly of astrocytes, in MERS patients (5). In addition, Tada et al. (2) speculated that intramyelinic edema and inflammatory cell infiltration were the causes.

Brain edema can be divided into two categories: vasogenic and cytotoxic. Vasogenic edema causes overall brain swelling due to openings in the blood–brain barrier, while cytotoxic edema causes cell swelling unrelated to the impermeability of the blood–brain barrier (21). Cytotoxic edema is also referred to as cellular edema, which damages glial cells and neurons (21, 22). In the present case, a diffusion-weighted MRI study showed hyperintensity in the SCC. Hypointensity areas in the ADC map MRI study were consistent with hyperintensity areas in the diffusion-weighted MRI study. It is widely accepted that these MRI signal patterns show cellular edema in the abnormal signal areas (23). Additionally, primary inflammation was not suggested in the areas at the onset of MERS. Thus, the initial occurrence was considered to be cellular edema caused by hypoglycemia, which subsequently caused cellular damage to astrocytes, as evidenced by the loss of GFAP-positive astrocytes and the loss of the myelin sheath of oligodendrocytes. Microglial reactions and minimal lymphocytic infiltration may occur secondary to cell damage.

Moreover, because glucose is associated with osmotic pressure, hypoglycemia can cause cell damage in tissues through cytotoxic edema. Osmotic demyelination syndrome occurs in the pons and extrapontine demyelination, including SCC (24, 25). Although we quickly replenished glucose when hypoglycemia was detected, it is possible that the rapid fluctuations in blood glucose affected osmotic pressure and demyelinated brain cells, leading to MERS. Regarding the specific localization of SSC for cytotoxic edema, a specific feature in SCC was presumed to be relatively higher tissue myelin water in the SCC (13, 26). Taken together, cytotoxic edema, independent of the etiology, may cause MERS. Previous pathophysiology was speculated in the absence of pathological findings; we believe that our report will help clarify the pathogenesis of MERS. Starkey (9) suggested the term of “the central pathogenesis of cytotoxic lesions of the corpus callosum (CLOCCS)” from the findings of the cytotoxic edema by MRI, which is the synonym of MERS. Now that we have clarified that cytotoxic edema could be the central pathology of MERS, the recently proposed term CLOCCS could be applicable to this syndrome as well.

As a limitation, we were unable to obtain pathology during the period of hyperintensity on MRI. Therefore, this may be a pathology at a time when hyperintensity has disappeared and may not reflect a pathology at a time when the condition is more aggressive. In addition, MRI studies were not sufficient to reveal the pathophysiological involvement of intramyelinic edema and inflammatory cell infiltration. For instance, diffusion-tensor imaging, quantitative susceptibility mapping, and R2* relaxometry analyses might be useful for these interpretations (27, 28), but we were unable to obtain these images. Moreover, we did not process pathology specimens for electron microscopy. If we could have provided the findings from the electron microscope, we would have been able to present more attractive content.

In conclusion, we revealed that the pathology of MERS was mainly caused by demyelination and cytotoxic edema.

## Data availability statement

The original contributions presented in the study are included in the article/supplementary material, further inquiries can be directed to the corresponding authors.

## Ethics statement

Ethical review and approval was not required for the study on human participants in accordance with the local legislation and institutional requirements. The participants provided their written informed consent to participate in this study. Written informed consent was obtained from the individual(s) for the publication of any potentially identifiable images or data included in this article.

## Author contributions

MH: Writing – original draft. MU: Writing – original draft. KojH: Conceptualization, Supervision, Writing – review & editing. EK: Supervision, Writing – review & editing. S-iA: Data curation, Writing – review & editing. AS: Data curation, Writing – review & editing. YN: Data curation, Writing – review & editing. RA: Data curation, Writing – review & editing. MS: Data curation, Writing – review & editing. TM: Data curation, Writing – review & editing. HH: Data curation, Writing – review & editing. KouH: Data curation, Writing – review & editing. YK: Data curation, Writing – review & editing.
